# 1,1′-(Ethane-1,2-di­yl)bis­(indoline-2,3-dione)

**DOI:** 10.1107/S1600536810018957

**Published:** 2010-06-05

**Authors:** Yao Wang, Sheng-Li Cao, Chong-Qing Wan, Jing-Li Yuan

**Affiliations:** a Department of Chemistry, Capital Normal University, Beijing 100048, People’s Republic of China

## Abstract

The mol­ecule of the title compound, C_18_H_12_N_2_O_4_, is situated on a crystallographic centre of symmetry. The mol­ecule has a zigzag structure, with two parallel symmetry-related indoline-2,3-dione fragments linked by an ethyl­ene group at each N atom. In the crystal, the mol­ecules stack in columns along the *b* axis. There are two such columns in the structure. The mol­ecules within each column are parallel; however, the mol­ecules in the two columns differ in the respective orientation of the indoline-2,3-dione fragments. In one column, they are approximately parallel to (112), while in the other they are approximately parallel to (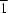
12). The inter­planar angle between the indoline-2,3-dione fragments in the two columns is 80.83 (3)°. The mol­ecules within each column are related by mutual displacement of their centres of symmetry, that is (0, ±1/2, ±1/2). The packing between the mol­ecules is provided by weak inter­actions only, *viz*. C—H⋯O hydrogen bonds and π–π [centroid–centroid distance = 3.8745 (8) Å] and C=O⋯π inter­actions.

## Related literature

For the biological and pharmacological activity of 1,2-bis­[(indolin-2,3-dion)-1-yl]ethane and its analogues, see: Breinholt *et al.* (1996 ▶); Norman (1996 ▶); Rajopadhye & Popp (1988 ▶). For details of the synthesis, see: Hyatt *et al.* (2007 ▶). For the melting point, see: Schmidt *et al.* (2008 ▶)·For a description of the Cambridge Structural Database, see: Allen (2002 ▶).
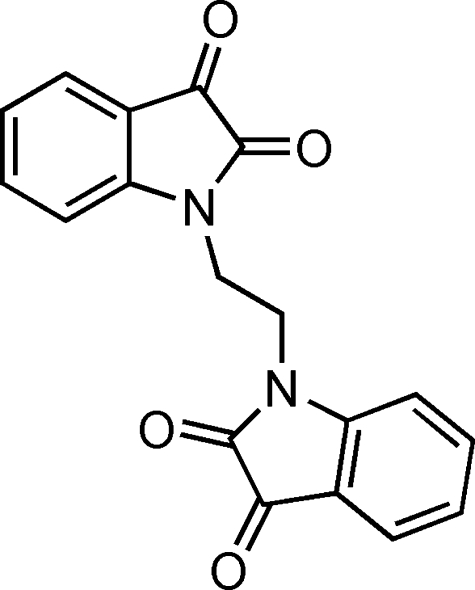


## Experimental

### 

#### Crystal data

C_18_H_12_N_2_O_4_
*M* *_r_* = 320.30
Monoclinic, 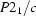

*a* = 12.2572 (3) Å
*b* = 5.2314 (1) Å
*c* = 12.5122 (3) Å
β = 115.747 (1)°
*V* = 722.66 (3) Å^3^
*Z* = 2
Mo *K*α radiation
μ = 0.11 mm^−1^
*T* = 296 K
0.45 × 0.32 × 0.25 mm

#### Data collection

Bruker APEXII CCD area-detector diffractometer
Absorption correction: multi-scan (*SADABS*; Bruker, 2007 ▶) *T*_min_ = 0.723, *T*_max_ = 0.893
13976 measured reflections
1714 independent reflections
1496 reflections with *I* > 2σ(*I*)
*R*_int_ = 0.028

#### Refinement

*R*[*F* ^2^ > 2σ(*F* ^2^)] = 0.040
*wR*(*F* ^2^) = 0.102
*S* = 1.05
1714 reflections
110 parameters
H-atom parameters constrained
Δρ_max_ = 0.19 e Å^−3^
Δρ_min_ = −0.16 e Å^−3^

### 

Data collection: *APEX2* (Bruker, 2007 ▶); cell refinement: *APEX2* and *SAINT* (Bruker, 2007 ▶); data reduction: *SAINT*; program(s) used to solve structure: *SHELXS97* (Sheldrick, 2008 ▶); program(s) used to refine structure: *SHELXL97* (Sheldrick, 2008 ▶); molecular graphics: *SHELXTL* (Sheldrick, 2008 ▶); software used to prepare material for publication: *SHELXTL* and *PLATON* (Spek, 2009 ▶).

## Supplementary Material

Crystal structure: contains datablocks I, global. DOI: 10.1107/S1600536810018957/fb2183sup1.cif

Structure factors: contains datablocks I. DOI: 10.1107/S1600536810018957/fb2183Isup2.hkl

Additional supplementary materials:  crystallographic information; 3D view; checkCIF report

## Figures and Tables

**Table 1:** Hydrogen-bond geometry (Å, °)

*D*—H⋯*A*	*D*—H	H⋯*A*	*D*⋯*A*	*D*—H⋯*A*
C9—H9*A*⋯O1^i^	0.97	2.47	3.262 (2)	139

Symmetry code: (i) 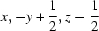.

**Table 2:** C=O⋯π inter­actions (Å, °) *Cg*1 and *Cg*2 are the centroids of the N1,C1,C6–C8 pyrrole and C1—C6 benzene rings, respectively.

C=O⋯*Cg*	O⋯*Cg*	C⋯*Cg*	C=O⋯*Cg*
C8—O2⋯*Cg*1	3.8207 (12)	4.4046 (12)	111.34 (10)
C8—O2⋯*Cg*1	3.6269 (15)	4.6449 (17)	142.86 (10)
C8—O2⋯*Cg*2	3.5874 (14)	3.5278 (14)	77.47 (9)

Symmetry codes: (i) 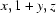; (ii) 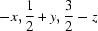; (iii) 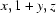.

**Fig. 1.**
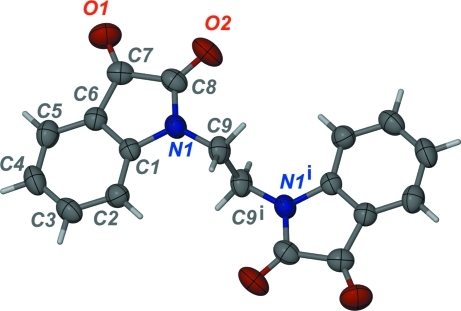
The title molecule with the atomic numbering scheme. The displacement ellipsoids are shown at the 50% probability level. Symmetry code: (i): -x, -y, -z+1.

**Fig. 2.**
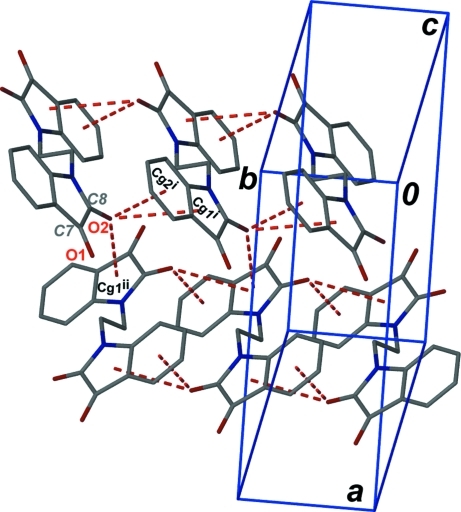
The column motifs of the molecules of the title compound. The π-electron ring···π-electron ring interactions are shown as a red dashed lines. Cg1, Cg2 are the centroids of the N1-C1-C6-C7-C8 (pyrrole) and C1-C2-C3-C4-C5-C6 (benzene) rings, respectively. Symmetry codes: (i): x, 1+y, z; (ii): -x, 1/2+y, 3/2-z.

## supplementary crystallographic information

### Comment

Isatins present a wide range of important biological and pharmacological activities. Fungicide (Breinholt *et al.*, 1996), antianxiety (Norman, 1996; Breinholt *et al.*, 1996) and anticonvulsant ones (Rajopadhye & Popp, 1988) are among them. In particular, the title compound, 1,2-bis[(indolin-2,3-dion)-1-yl]ethane, and related 1,1-bis{4-[(2,3-dioxoindolin-1-yl)methyl]phenyl}methane and 1-(3,4-dichlorobenzyl)indoline-2,3-dione (Hyatt *et al.*, 2007) have been considered as potent and selective carboxylesterase inhibitors (Hyatt *et al.*, 2007). Herein, we report the structure of the title compound.

The centrosymmetric molecule takes a zigzag fashion, with two symmetric parallel indoline-2,3-dione fragments being linked by the ethylene group to the N atoms (Fig. 1). There are only weak intermolecular interactions in the structure: a π-electron—π-electron ring interaction between N1\C1\C6\C7\C8 (pyrrole) and C1\C2\C3\C4\C5\C6 (benzene) rings (symmetry code: *x*, *y*+1, *z*) with the distance between the respective centroids equal to 3.8745 (8) Å. A C-H···O bond is given in Tab. 1 while C═O···π-electron ring interactions are listed in Tab. 2.

The distance C7-C8 (1.5554 (19) Å) corresponds well to the pertinent distances previously observed in well determined structures with the indoline-2,3-dione fragment. The search in the Cambridge Structural Database (Allen, 2002; Cambridge Structural Database, version 5.31 and addenda up to 26 February 2010) yielded 12 hits with structures determined with R_val_<0.05. The corresponding extremal distances from this search equalled to 1.531 and 1.578 Å for JOBDEG and NAQRAY, respectively.

### Experimental

A mixture of indoline-2,3-dione (1.47 g, 10 mmol), 1,2-dibromoethane (5.64 g, 30 mmol) and K_2_CO_3_ (2 g, 14.5 mmol) in *N,N*-dimethylformamide (20 ml) was heated at 100-120 °C for 3 h. After cooling to room temperature, the reaction mixture was poured into 0°C water (100 ml). The resulting precipitate was separated by filtration, dried in air and then it was purified by column chromatography on a silica gel with dichloromethane/methanol = 95:5, v/v, as an eluent. The precipitate included the prevailing product 1-(2-bromoethyl)indoline-2,3-dione (*R*_f_ = 0.77,  m.p. 131-132°C; yield 60.9%) as well as the the title product 1,2-bis[(indolin-2,3-dion)-1-yl]ethane (*R*_f_ = 0.64, m.p. 296-297°C; yield 13.1%). The prevailing product, 1-(2-bromoethyl)indoline-2,3-dione, has been determined by a mass spectrometric analysis while its melting point (131-132°C) corresponded to 131°C reported by Schmidt *et al.* (2008). The orange crystals of the title compound that measured 0.40 × 0.30 × 0.20 mm on average were obtained by slow evaporation from the solution of dichloromethane *N,N*-dimethylformamide 50:50 (v/v).

### Refinement

All the H atoms were discernible in the difference electron density maps. Nevertheless, the hydrogen atoms were placed into the idealized positions and allowed to ride on the carrier atoms, with C—H = 0.93 and 0.97 Å for aryl and methylene hydrogens, respectively. *U*_iso_(H) = 1.2*U*_eq_(C)_aryl_/_methylene_.

### Crystal data

**Table:**

C_18_H_12_N_2_O_4_	*F*(000) = 332
*M**_r_* = 320.30	*D*_x_ = 1.472 Mg m^−^^3^
Monoclinic, *P*2_1_/*c*	Melting point = 569–570 K
Hall symbol: -P 2ybc	Mo *K*α radiation, λ = 0.71073 Å
*a* = 12.2572 (3) Å	Cell parameters from 243 reflections
*b* = 5.2314 (1) Å	θ = 1.8–27.2°
*c* = 12.5122 (3) Å	µ = 0.11 mm^−^^1^
β = 115.747 (1)°	*T* = 296 K
*V* = 722.66 (3) Å^3^	Block, orange
*Z* = 2	0.45 × 0.32 × 0.25 mm

### Data collection

**Table:**

Bruker APEXII CCD area-detector diffractometer	1714 independent reflections
Radiation source: fine-focus sealed tube	1496 reflections with *I* > 2σ(*I*)
graphite	*R*_int_ = 0.028
ω scans	θ_max_ = 27.9°, θ_min_ = 1.8°
Absorption correction: multi-scan (*SADABS*; Bruker, 2007)	*h* = −16→16
*T*_min_ = 0.723, *T*_max_ = 0.893	*k* = −6→6
13976 measured reflections	*l* = −16→16

### Refinement

**Table:**

Refinement on *F*^2^	Secondary atom site location: difference Fourier map
Least-squares matrix: full	Hydrogen site location: difference Fourier map
*R*[*F*^2^ > 2σ(*F*^2^)] = 0.040	H-atom parameters constrained
*wR*(*F*^2^) = 0.102	*w* = 1/[σ^2^(*F*_o_^2^) + (0.0422*P*)^2^ + 0.1818*P*] where *P* = (*F*_o_^2^ + 2*F*_c_^2^)/3
*S* = 1.05	(Δ/σ)_max_ < 0.001
1714 reflections	Δρ_max_ = 0.19 e Å^−^^3^
110 parameters	Δρ_min_ = −0.16 e Å^−^^3^
0 restraints	Extinction correction: *SHELXL97* (Sheldrick, 2008), Fc^*^=kFc[1+0.001xFc^2^λ^3^/sin(2θ)]^-1/4^
24 constraints	Extinction coefficient: 0.060 (5)
Primary atom site location: structure-invariant direct methods	

### Special details

**Table:**

Geometry. All esds (except the esd in the dihedral angle between two l.s. planes) are estimated using the full covariance matrix. The cell esds are taken into account individually in the estimation of esds in distances, angles and torsion angles; correlations between esds in cell parameters are only used when they are defined by crystal symmetry. An approximate (isotropic) treatment of cell esds is used for estimating esds involving l.s. planes.
Refinement. Refinement of *F*^2^ against ALL reflections. The weighted *R*-factor wR and goodness of fit *S* are based on *F*^2^, conventional *R*-factors *R* are based on *F*, with *F* set to zero for negative *F*^2^. The threshold expression of *F*^2^ > σ(*F*^2^) is used only for calculating *R*-factors(gt) etc. and is not relevant to the choice of reflections for refinement. *R*-factors based on *F*^2^ are statistically about twice as large as those based on *F*, and *R*- factors based on ALL data will be even larger.

### Fractional atomic coordinates and isotropic or equivalent isotropic displacement parameters (Å^2^)

**Table:**

	*x*	*y*	*z*	*U*_iso_*/*U*_eq_	
C1	0.22945 (10)	−0.0885 (2)	0.68434 (10)	0.0390 (3)	
C2	0.26127 (11)	−0.2721 (3)	0.62433 (12)	0.0474 (3)	
H5	0.2193	−0.2905	0.5425	0.057*	
C3	0.35869 (12)	−0.4288 (3)	0.69112 (14)	0.0539 (4)	
H4	0.3818	−0.5550	0.6527	0.065*	
C4	0.42235 (12)	−0.4035 (3)	0.81260 (14)	0.0566 (4)	
H3	0.4875	−0.5110	0.8545	0.068*	
C5	0.38964 (12)	−0.2189 (3)	0.87211 (12)	0.0526 (3)	
H2	0.4318	−0.2010	0.9540	0.063*	
C6	0.29262 (11)	−0.0609 (2)	0.80716 (11)	0.0429 (3)	
C7	0.23681 (12)	0.1498 (3)	0.84203 (12)	0.0478 (3)	
C8	0.13013 (12)	0.2405 (2)	0.72484 (12)	0.0488 (3)	
C9	0.04837 (11)	0.1014 (3)	0.51355 (11)	0.0484 (3)	
H9A	0.0900	0.0746	0.4639	0.058*	
H9B	0.0108	0.2689	0.4955	0.058*	
N1	0.13504 (9)	0.0917 (2)	0.63726 (9)	0.0448 (3)	
O2	0.05930 (10)	0.4097 (2)	0.71384 (11)	0.0696 (4)	
O1	0.26316 (11)	0.2447 (2)	0.93787 (9)	0.0691 (4)	

### Atomic displacement parameters (Å^2^)

**Table:**

	*U*^11^	*U*^22^	*U*^33^	*U*^12^	*U*^13^	*U*^23^
C1	0.0363 (5)	0.0370 (6)	0.0449 (6)	−0.0013 (5)	0.0187 (5)	0.0035 (5)
C2	0.0446 (6)	0.0499 (8)	0.0498 (7)	−0.0001 (5)	0.0223 (5)	−0.0031 (6)
C3	0.0506 (7)	0.0464 (8)	0.0736 (9)	0.0055 (6)	0.0352 (7)	0.0004 (7)
C4	0.0436 (7)	0.0509 (8)	0.0731 (9)	0.0099 (6)	0.0232 (7)	0.0159 (7)
C5	0.0471 (7)	0.0550 (8)	0.0488 (7)	−0.0009 (6)	0.0145 (6)	0.0106 (6)
C6	0.0442 (6)	0.0403 (6)	0.0449 (6)	−0.0024 (5)	0.0200 (5)	0.0030 (5)
C7	0.0573 (7)	0.0441 (7)	0.0500 (7)	−0.0051 (6)	0.0306 (6)	−0.0006 (6)
C8	0.0546 (7)	0.0393 (7)	0.0623 (8)	0.0022 (6)	0.0344 (6)	0.0042 (6)
C9	0.0438 (6)	0.0483 (7)	0.0494 (7)	0.0030 (6)	0.0169 (6)	0.0149 (6)
N1	0.0425 (5)	0.0424 (6)	0.0477 (6)	0.0057 (4)	0.0179 (5)	0.0044 (5)
O2	0.0796 (7)	0.0511 (6)	0.0934 (9)	0.0219 (5)	0.0518 (7)	0.0109 (6)
O1	0.0868 (8)	0.0726 (8)	0.0564 (6)	−0.0037 (6)	0.0390 (6)	−0.0127 (5)

### Geometric parameters (Å, °)

**Table:**

C1—C2	1.3757 (17)	C5—H2	0.9300
C1—C6	1.3958 (17)	C6—C7	1.4605 (18)
C1—N1	1.4081 (15)	C7—O1	1.2051 (16)
C2—C3	1.3892 (19)	C7—C8	1.5554 (19)
C2—H5	0.9300	C8—O2	1.2053 (16)
C3—C4	1.381 (2)	C8—N1	1.3665 (17)
C3—H4	0.9300	C9—N1	1.4486 (16)
C4—C5	1.381 (2)	C9—C9^i^	1.515 (3)
C4—H3	0.9300	C9—H9A	0.9700
C5—C6	1.3847 (18)	C9—H9B	0.9700
			
C2—C1—C6	121.32 (11)	C1—C6—C7	107.40 (11)
C2—C1—N1	127.98 (11)	O1—C7—C6	130.57 (14)
C6—C1—N1	110.70 (11)	O1—C7—C8	124.38 (13)
C1—C2—C3	117.20 (12)	C6—C7—C8	105.05 (10)
C1—C2—H5	121.4	O2—C8—N1	127.39 (14)
C3—C2—H5	121.4	O2—C8—C7	126.91 (13)
C4—C3—C2	122.18 (13)	N1—C8—C7	105.69 (11)
C4—C3—H4	118.9	N1—C9—C9^i^	110.73 (13)
C2—C3—H4	118.9	N1—C9—H9A	109.5
C3—C4—C5	120.21 (13)	C9^i^—C9—H9A	109.5
C3—C4—H3	119.9	N1—C9—H9B	109.5
C5—C4—H3	119.9	C9^i^—C9—H9B	109.5
C4—C5—C6	118.51 (13)	H9A—C9—H9B	108.1
C4—C5—H2	120.7	C8—N1—C1	111.12 (10)
C6—C5—H2	120.7	C8—N1—C9	124.73 (11)
C5—C6—C1	120.58 (12)	C1—N1—C9	123.94 (11)
C5—C6—C7	132.02 (12)		
			
C6—C1—C2—C3	0.09 (18)	O1—C7—C8—O2	−1.6 (2)
N1—C1—C2—C3	−179.95 (12)	C6—C7—C8—O2	179.12 (13)
C1—C2—C3—C4	−0.3 (2)	O1—C7—C8—N1	177.33 (13)
C2—C3—C4—C5	0.4 (2)	C6—C7—C8—N1	−1.95 (13)
C3—C4—C5—C6	−0.3 (2)	O2—C8—N1—C1	−179.26 (13)
C4—C5—C6—C1	0.07 (19)	C7—C8—N1—C1	1.81 (13)
C4—C5—C6—C7	−179.40 (13)	O2—C8—N1—C9	−4.4 (2)
C2—C1—C6—C5	0.03 (18)	C7—C8—N1—C9	176.68 (11)
N1—C1—C6—C5	−179.94 (11)	C2—C1—N1—C8	179.03 (12)
C2—C1—C6—C7	179.61 (11)	C6—C1—N1—C8	−1.01 (14)
N1—C1—C6—C7	−0.36 (13)	C2—C1—N1—C9	4.11 (19)
C5—C6—C7—O1	1.7 (2)	C6—C1—N1—C9	−175.93 (11)
C1—C6—C7—O1	−177.84 (14)	C9^i^—C9—N1—C8	−95.50 (17)
C5—C6—C7—C8	−179.10 (13)	C9^i^—C9—N1—C1	78.73 (17)
C1—C6—C7—C8	1.38 (13)		

Symmetry codes: (i) −*x*, −*y*, −*z*+1.

### Hydrogen-bond geometry (Å, °)

**Table:**

*D*—H···*A*	*D*—H	H···*A*	*D*···*A*	*D*—H···*A*
C9—H9A···O1^ii^	0.97	2.47	3.262 (2)	139

Symmetry codes: (ii) *x*, −*y*+1/2, *z*−1/2.

### Table 2 C═O···π interactions (Å, °)

Cg1 and Cg2 are the centroids of the N1,C1,C6–C8 pyrrole and C1—C6 benzene rings, respectively.

**Table:**

C═O···Cg	O···Cg	C···Cg	C═O···Cg
C8—O2···Cg1	3.8207 (12)	4.4046 (12)	111.34 (10)
C8—O2···Cg1	3.6269 (15)	4.6449 (17)	142.86 (10)
C8—O2···Cg2	3.5874 (14)	3.5278 (14)	77.47 (9)

Symmetry codes: (i) *x*, 1+*y*, *z*; (ii) -x, 1/2+*y*, 3/2-*z*; (iii) *x*, 1+*y*, *z*.
